# Widespread transmission in diverse ecotypes challenges visceral leishmaniasis control in East Africa

**DOI:** 10.1101/2025.10.01.25337091

**Published:** 2025-10-03

**Authors:** Eva Iniguez, Mercy Tuluso, Steve Kiplagat, Araya Gebresilassie, Esayas Aklilu, Olivia Battistoni, Johnstone Ingonga, John Mark Makwatta, Mohamed Alamin, Osman Dakien, Alphine Chebet, Esther Kaunda, Patrick Huffcutt, Serena Doh, Pedro Cecilio, Galgallo Bonaya, Claudio Meneses, Tiago D. Serafim, Myrthe Pareyn, Mohamed Osman, Eltahir A. G. Khalil, Omran F. Osman, Brima M. Younis, Guofa Zhou, Jesus G. Valenzuela, Dan K. Masiga, Ahmed M. Musa, Asrat Hailu, Abhay Satoskar, Shaden Kamhawi, Damaris Matoke-Muhia

**Affiliations:** 1 Vector Molecular Biology Section, Laboratory of Malaria and Vector Research, National Institute of Allergy and Infectious Diseases, National Institutes of Health, Rockville, MD, USA; 2 Centre for Biotechnology Research and Development, Kenya Medical Research Institute, Nairobi, Kenya; 3 International Centre of Insect Physiology and Ecology (*icipe*), Nairobi, Kenya; 4 Department of Zoological Sciences, College of Natural and Computational Sciences, Addis Ababa University, Addis Ababa, Ethiopia; 5 Vector Biology and Control Research Unit, Aklilu Lemma Institute of Pathobiology, Addis Ababa University, Addis Ababa, Ethiopia; 6 Departments of Pathology and Microbiology, Wexner Medical Center, The Ohio State University, Columbus, OH, USA; 7 Department of Zoology, Faculty of Science, University of Khartoum, Khartoum, Sudan; 8 Kala Zara Research Center, Faculty of Medicine and Health Science, University of Gedaref, Gedaref, Sudan; 9 Vector Biology Section, Laboratory of Malaria and Vector Research, National Institute of Allergy and Infectious Diseases, National Institutes of Health, Rockville, MD, USA; 10 Department of Clinical Sciences, Institute of Tropical Medicine Antwerp, Belgium; 11 York Biomedical Research Institute, University of York, York, UK; 12 Program in Public Health, University of California at Irvine, Irvine, CA, USA; 13 Department of Clinical Pathology and Immunology, Institute of Endemic Diseases, University of Khartoum, Khartoum, Sudan; 14 College of Health Sciences, Addis Ababa University, Addis Ababa, Ethiopia

**Keywords:** East Africa, visceral leishmaniasis, rK39 seroprevalence, ecotype, microhabitat, breeding sites, blood meal analysis, *Leishmania*-infected sand flies, transmission

## Abstract

East Africa is emerging as the global hot spot of visceral leishmaniasis (VL), yet efforts to eliminate it are hindered by substantial knowledge gaps in its ecoepidemiology. Here, we report on the high prevalence of *Leishmania* infection in *Phlebotomus orientalis* in Marsabit county, Kenya (3.9%), and Gedaref state, Sudan (3.6%), where this species comprised 99.8% (n = 1185) and 100% (n = 1350) of captured *Phlebotomus* females, respectively. In Aba Roba, Ethiopia, *Ph. martini* accounted for 99% of 184 collected *Phlebotomus* females and had a lower infection rate of 1.5%. *Ph. orientalis* and *Ph. martini* exhibited different habitat and feeding preferences. While *Ph. orientalis* was abundant in diverse peridomestic and sylvatic microhabitats, *Ph. martini* was predominantly collected from termite hills. Moreover, *Ph. orientalis* primarily fed on humans and less on domestic and sylvatic animals. In contrast, *Ph. martini* exhibited zoophagic behavior, mostly feeding on cows and ovis. Widespread transmission of *Leishmania* in our study sites is supported by high rK39 seroprevalence in both Kenya (20.88%) and Sudan (7.36%). An observed greater prevalence of antibodies to rK39 in individuals living near than away from VL cases in both Kenya (21.68% versus 16.51%, *P* = 0.03) and Sudan (10.03% versus 2.05%, *P* = 0.02) demonstrated that proximity to a VL case carries an increased risk of infection. Our findings highlight the need for a targeted site-specific elimination strategy that accounts for the intensity, diversity, and complexity of VL transmission in today’s East Africa.

## Introduction

East Africa is currently the epicenter of global transmission of visceral leishmaniasis (VL), a fatal neglected disease ranked second after malaria in mortality rates^(1)^. VL in East Africa is epidemiologically and clinically diverse, caused by genetically distinct *Leishmania donovani* strains^(2–4)^, and transmitted by several sand fly vector species including *Phlebotomus orientalis, Ph. martini,* and *Ph. celiae* that have divergent ecologies and bionomics^(5–8)^. VL cases have recently surged in East Africa, emerging in new foci that disproportionally affected Kenya, Sudan, and Ethiopia, accounting for more than 65% of cases worldwide, out of which ~50% are children under 15 years old^(1, 9)^.

VL in East Africa had initially been targeted for control rather than elimination due to significant knowledge gaps concerning disease transmission^(10)^. In June 2024, the World Health Organization launched a framework to eliminate VL as a public health concern from East Africa by 2030^(11)^. Elimination is proposed through early diagnosis and treatment, integrated vector control management, and disease surveillance^(11)^. At the country level, the target for elimination was defined as <1% case-fatality rate due to primary VL, 90% decline in new VL cases, absence of death due to VL in children by 2030, and 100% detection and treatment referral of VL-HIV and PKDL patients^(11)^. However, East Africa faces major challenges to VL elimination. Treatment differs between countries, and it is often toxic and lengthy, with low compliance and poor access to care facilities^(5, 11–13)^. Moreover, the serological test for *Leishmania* diagnosis, the rK39 rapid test, has a decreased sensitivity and specificity in regions of East Africa, particularly in HIV-VL co-infected patients^(14–16)^. In addition, significant knowledge gaps in ecoepidemiology, vector biology, and nature of reservoirs need to be addressed for a successful implementation of an elimination campaign in East Africa,^(5, 12)^ where there are no current effective integrated vector control programs^(11)^. Though *Ph.* orientalis, *Ph. martini*, and *Ph. celiae* sand flies have been found in both peridomestic and sylvatic niches in strong association with *Acacia* and *Balanites* woodlands, vertisol (black cotton soil), and termite hills^(17–20)^, linking these microhabitats to VL transmission in the region^(19, 21–24)^, confirmation of their epidemiological relevance and their potential as resting and breeding sites for these vectors^(20, 25)^ needs to be demonstrated. Additionally, while VL caused by *L. donovani* is considered anthroponotic, the opportunistic feeding behavior reported for *Ph. orientalis* together with high transmission rates reported in relatively scarcely populated areas of East Africa, points to the potential presence of an animal reservoir^(17, 24, 26–28)^. Additionally, we need to determine the role of asymptomatic individuals as infectious reservoirs for sand flies^(29–31)^. Further hindering control of VL in East Africa is the presence of semi-nomadic populations, such as pastoralists and farmers living in temporary shelters^(12)^, and populations displaced by conflict^(32, 33)^, that are at a higher risk of contracting VL^(32–34)^, complicating the epidemiological landscape.

In this study, we report the prevalence of *Leishmania*-infected sand flies from diverse microhabitats in endemic study sites in Kenya, Sudan, and Ethiopia. We also establish widespread transmission through the high rate of rK39 seroprevalence in Marsabit county, Kenya, and Gedaref state, Sudan, where *Ph. orientalis* is the primary vector. Altogether, this study provides current information on VL transmission dynamics in East Africa.

## Methods

### Ethics approval

The study was approved by the Scientific Research Unit, KEMRI, and licensed by The National Commission for Science, Technology and Innovation (clinical protocol # KEMRI/SERU/CBRD/249/4634) and by the State Ministry of Health and Social Development at Gedaref state, Sudan ethical committee (clinical protocol # UG/EC/3/2023) in Sudan. Both clinical protocols were translated into local languages. Communal consent was obtained by meeting with community leaders. Written consent was obtained from adults or guardians of minors before blood collection. Verbal consent was acquired from homeowners before entomological collections.

### Study sites

We selected study sites with stable VL transmission in Kenya, Sudan, and Ethiopia. The study sites were chosen to encompass features common to VL ecotypes in East Africa.

#### Kenya

Marsabit county has a population of 459,785 people^(35)^, and has experienced sporadic outbreaks since 2014^(36, 37)^. It has an arid and semi-arid tropical savanna climate with an average temperature ranging between 15°C and 26°C. Average precipitation is about 128.24 mm per annum. Marsabit county has three climatic seasons: two dry seasons in January to February and June to July, short rains in March to May, and long rains in August to December ^(38)^. The communal free-range land is dominated by diverse *Acacia* and related trees, a wide variety of shrubs, and seasonal grass.

Inhabitants of Marsabit county are mainly nomadic pastoralists belonging to one of fourteen Cushitic-speaking ethnic groups. Camel, goat, sheep, donkeys, and cattle are major domestic animals kept as a source of income. The community moves with their domestic animals from semi-permanent (manyatta) houses to temporary houses (foras) for a period of up to six months in search of pasture and water. We investigated three locations: Laisamis, Logo Logo, and Karare, in Laisamis sub-county, Marsabit county.

#### Sudan

Gedaref state has an urban structure with well-established villages. The region is a flat plain, with small, scattered hills and flowing watercourses, and the climate is tropical. The rainy season spans June to October with an annual rainfall of 400–1400 mm. The natural vegetation is a savanna woodland with *Acacia* and *Balanites* trees, and the principal soil type is black cotton vertisol. Agriculture is the main livelihood, with sorghum, sesame, and millet as major crops. Goats, sheep, donkeys, and cattle are major domestic animals kept as a source of income. Most people are subsistence farmers or engage in small animal husbandry. High demand for manual labor attracts seasonal workers from within Sudan or bordering Ethiopia during the rainy season. In a recent study, a high VL prevalence of 27.6% was reported among Dinder National Park wildlife soldiers^(39)^. Dinder National Park falls between the Sahel of Gedaref state and Ethiopian Highlands and is home to several species of large and small mammals, such as lions, gazelle, monkeys, bats and reptiles. We investigated the villages of Kunaynah, bordering north Ethiopia, and Umslala-Houng, closest to Dinder National Park, in Gedaref state.

#### Ethiopia

The Konso zone is a semi-arid rugged terrain with many hills, lowlands, and riverbeds. The area is generally semi-arid, with an annual rainfall of about 551 mm per year. The long rains last from February to May, followed by short rains in September and October. The mean annual temperature is 24°C. The predominant soil type is sandy. Dispersed homesteads and animal enclosures are scattered in lowlands or valleys, while villages are on top of large hills or highlands whose slopes lead to the valleys. Segen river flows through the valley providing water for irrigation and creating flat plains suitable for farming and animal herding. Even though there are no permanent human settlements, many families own farms in the valleys and spend a considerable number of nights in temporary shelters (foras) herding cattle, goats and sheep. The vegetation comprises a mixture of shrub and forests with diverse trees, including species of *Acacia* and *Balanites*. Castellated active or eroded shrinking termite hills^(7)^ are abundant in the area. Aba Roba communities mostly depend on subsistence farming on the cultivated terraced hillsides and flat plains of the Segen River. Sorghum, maize, sesame and sunflower are major crops. They also raise goats, sheep, and cattle, kept in enclosures at night. We investigated the villages of Galga and Goinada, 1180–1250 m above sea level, and Maira and Lahalaha, 830–885 m above sea level, in the Konso zone.

### Sand fly collection

In each country, sand fly sampling ecotypes included peridomestic (associated with humans) and sylvatic (away from humans) sampling microhabitats (Supplementary Figure 1). Ecotypes included indoors (inside manyatta/hut) and outdoors (outside manyatta/hut), temporary shelters or foras, animal enclosures, vegetation in association or not with animal burrows and vertisols, and termite hills. The study sites and the total number of CDC light traps used per collection varied in each country, but the same number of traps were placed per ecotype (Supplementary Table 1).

### Species identification of *Phlebotomus* sand flies

The head and the last three segments of each *Phlebotomus* sand fly were wet mounted for morphological identification. Spermatheca and the pharynx teeth were examined to distinguish the female species, while external genitalia were examined for identification of males using a combination of keys^(40, 41)^.

### Rotation of *Phlebotomus* male genitalia

During the first 16–24 h of the adult life of a male sand fly the external genitalia undergo a rotation to orient the claspers during mating. Thus, unrotated or partially rotated male genitalia can be used to identify newly emerged juveniles captured during their first night of activity as adults, as a proxy to identify sand fly breeding sites^(17, 20, 22)^. Under the microscope, we recorded the number of males with rotated, unrotated or partially rotated genitalia.

### Mitigation of sample contamination from the field to the bench

In the field, all female sand flies were dissected using dissecting pins that were first bleached (10%) and rinsed twice with water before each sand fly dissection. This step was critical to avoid contamination caused by carry over DNA from sample to sample. In the laboratory, three separate rooms were used to process and run the field samples by qPCR. Room one was used only for preparation of buffer and primer aliquots and loading of the qPCR master mix into the plate. Room two was used for extraction of nucleic acids of field specimens. Room three was used to load the DNA samples into the qPCR plate. Each plate included a standard curve, and DNA from an uninfected midgut and non-template negative controls. To avoid contamination all the instruments, benches and pipettes were continuously bleached (10%) and rinsed with water.

### Extraction of DNA from field-collected *Phlebotomus* female sand flies

Unfed and gravid midgut samples were individually collected in Ethiopia, and individual or pools of 5 midguts in Kenya. For Sudan, pools of 10 sand flies (whole bodies or midguts) were collected. Samples were stored in 50 μl of DNA/RNA Shield buffer (Zymo), and stored at room temperature, or 4°C if available. DNA was extracted from individual specimens of unfed and gravid sand flies using the Quick-DNA/RNA Miniprep Plus kits (Zymo) tissue protocol, with a proteinase K and tissue lysis digestion step of 1 h 56°C. DNA was extracted from pools of unfed and gravid sand flies using the Tissue DNA kit (Omega) according to the manufacturer’s instructions. For whole body pooled samples, we first macerated each using a plastic pestle, followed by chemical digestion with proteinase K and tissue lysis buffer for 2–6 h or until fully digested at 56°C.

Blood-fed sand flies were preserved individually as midguts/whole bodies on Whatman 903 Protein Saver cards (Cytica), or in up to 50 μl of DNA/RNA Shield buffer (Zymo). Briefly, field-collected blood fed midguts were individually punched from the Whatman cards using a 3 mm punch biopsy, and Proteinase K (20 μl), Lysis buffer (20 μl), and DNA/RNA shield (30 μl) were added to each punched sample and digested at 56°C for 2 to 4 h, with brief vortexing preformed every hour. Blood fed samples stored in DNA/RNA Shield buffer as midguts/whole bodies were first macerated using ceramic bashing beads (Roche) using the MagNAlyzer (Roche), followed by digestion for 24 h. DNA was extracted using the Quick-DNA/RNA Miniprep Plus kits (Zymo). All samples were eluted to a final volume of 100 μL and stored at −20/−80°C until further processing.

### Blood meal analysis by multiplex PCR

#### Standardization of a blood meal multiplex custom PCR panel

*Lutzomyia longipalpis* (Jacobina colony) was reared at the Laboratory of Malaria and Vector Research, NIAID, NIH insectary. Female sand flies (4–6 day old) were allowed to feed for 2 h on whole blood from various species through an artificial chicken skin membrane^(42)^ and collected 24 h post-feeding as positive controls. Blood meals included domestic and sylvatic hosts such as mouse and chicken (LMVR4 animal protocol); human (leftover samples collected for another study under IRB human protocol 000331-I); cow (Charles River Laboratories); dog, goat, sheep, and donkey (Innovative Research); hyrax and camel (donated by Dan Masiga, *icipe*).

A universal reverse primer previously reported^(43)^, with custom forward primers developed based on the mitochondrial *cytochrome b* region, highly conserved among vertebrates^(44)^, were used for several species of interest (Supplementary Table 2) . A human primer set based on the cytochrome c oxidase I mitochondrial region^(45)^, and the rodent (*Muroidea* superfamily) primer set targeting the mitochondrial d-loop region were used from previous published studies^(46)^. Briefly, DNA (40 ng, 10 μL) was mixed in a single tube as a multiplex reaction with all forward and reverse primers and the DreamTaq^™^ Hot Start Green PCR Master Mix (Thermo Scientific) to a final volume of 30 μL (Supplementary Table 2). PCR conditions were 95°C initial denaturation for 5 minutes, 35 cycles of 20 seconds at 95°C, 20 seconds at 58°C, 60 seconds at 72°C, and a final extension of 7 minutes at 72°C. PCR products were separated on a 2% agarose gel electrophoresis ran at 8 v/cm for 60 minutes. Two microliters of a 50 to 1000 base-pair ladder (Thermo Scientific) was used. Bands were visualized on a c600 Azure Biosystems imager.

#### Bloodmeal analysis for field-collected samples

PCR of DNA from field samples was performed following the same procedure and conditions described above and in Supplementary Table 2. The PCR products were run on a 2% agarose gel, cut and purified (Thermo Scientific). The PCR conditions were 94°C initial denaturation for 5 min, 35 cycles of 30 s at 94°C, 30 s at 55°C, 60 s at 72°C, and a final extension of 7 min at 72°C. For field samples that did not amplify against any of our custom-made targets, the DNA was amplified using a PCR against generic vertebrate primers (0.2 μM) for the *cytochrome b* gene region^(47)^. The resulting products were again purified and reamplified under the same conditions. Five microliters of each reamplified product were separated on a 2% agarose gel. The remaining product was purified using GeneJet PCR purification kit (Thermo Scientific) and sequenced (Eurofins Genomics). Sequences were aligned with reference genomes from the NCBI database to determine blood meal identity.

### *Leishmania* detection and quantification by qPCR

A standard curve was created for each different sample condition by spiking and uninfected sand fly midgut, a whole body, or a pool of whole bodies, with 10^5^ *L. donovani* (MHOM/SD/62/1S) parasites followed by DNA extraction, followed by a 10-fold serial dilution.

For unfed and gravid samples (processed individually or as a pool of 5–10 sand flies), we used qPCR to amplify the kinetoplast minicircle from *Leishmania* parasites using JW11 and JW12 primers^(42)^ in a 25 μL reaction volume containing SYBR green (PerfeCTA SYBR Green Fast Mix or FIREPol Master Mix Ready to Load). PCR conditions were 95°C initial denaturation for 3 min with 49 cycles of 10 s at 95°C and 30 s at 55°C. A probe-based qPCR was used for individually blood fed sand flies as described^(42)^. All field samples were run in duplicate (unfed/gravid) or triplicate (blood fed) using 40 ng of DNA template. A standard curve (of serially diluted parasite-spiked midguts), and negative (uninfected laboratory-reared sand flies) and positive (*Leishmania donovani*-infected laboratory-reared sand flies) controls were included in each plate. Melting curve analysis of field-samples were compared to the melting curve from positive and negative controls. Samples considered positive for *Leishmania* kDNA by qPCR were re-run to confirm positive amplification.

### Human dried blood spot collection

All reported VL cases to the Ministry of Health were diagnosed and treated at reference hospitals according to national guidelines^(11, 48)^. Males and females were included in our study. A total of 752 participants were enrolled from Laisamis and Karare, Marsabit county, Kenya, and 440 participants were enrolled from Kunaynah village, Gedaref state, Sudan. Blood was collected by finger prick using a lancet, spotted on a Whatman 903 protein saver card (Cytiva), and preserved at room temperature.

### *Leishmania* rK39 ELISA from dried blood spots

ELISA plates (Thermo Fisher Scientific) were coated overnight at 4°C with 25 ng/well of rK39 antigen (donated by Steve Reed from the Infectious Disease Research Institute, now the Access to Advanced Health Institute). Plates were blocked with 200 μl diluted in Tis-Bris Saline 0.05% Tween 20 (TBST) with 20% donor equine serum (DES; Cytiva), and incubated for 1 h at room temperature. In parallel, dried blood spots (DBSs) were eluted with 300 μl of PBS 0.05% Tween 20 (Sigma), and incubated for 4 h shaking at room temperature or overnight at 4°C. After blocking, 25 μl of the eluted DBS sample was diluted in TBST/5% DES (1:4 dilution) to a total volume of 100 μl/well in duplicates. Plates were incubated for 1 h at room temperature, followed by 6 washes with TBST. One hundred microliters of secondary antibody, [Goat anti-human IgG (H+L) Alkaline phosphatase conjugated; Sigma], diluted in TBST/5% DES was added to each well. After 1 h of incubation, the plates were washed 12 times with TBST. Lastly, 100 μl of substrate-p-nitrophenyl phosphate liquid (Sigma) was added to each well and data was collected 1 h after addition of the substrate at a 405 nm wavelength. Non-endemic negative controls obtained from individuals living in Nairobi, Kenya were included in each plate.

### Statistical analysis

Chi-square goodness of fit test was performed for entomology analysis, followed by Post-hoc z-score analysis with Bonferroni correction to compare ecotypes within each country. A z-score ≥ 2.69 was considered significant. ELISA data was normalized using the plate cutoff calculated by the mean optical density (O.D.) of non-endemic negative controls + 2 SD, followed by log transformation. After plate normalization, samples with an O.D. ≤ 0.04 were considered negative and statistical comparisons were calculated by the Mann-Whitney test. A *P* value of ≤ 0.05 was considered significant.

## Results

### Selection of study sites in Kenya, Sudan, and Ethiopia

**Figure 1.**
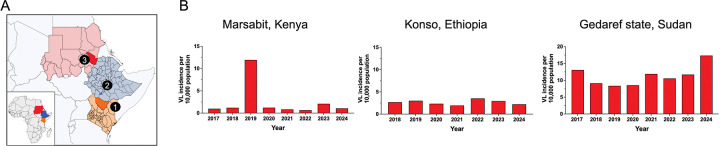
Study sites and incidence of visceral leishmaniasis (A,B) Map of East Africa showing the location of study sites. 1, Marsabit ; 2, Konso; 3, Gedaref state. (B) Visceral leishmaniasis (VL) cases per 10,000 population over the past seven years in Marsabit county, Kenya; Konso, Ethiopia; and Gedaref state, Sudan. Incidence data are based on Ministry of Health reports from each country. Maps at QGIS software QGIS shapefile, https://open.africa/dataset/?tags=Shapefiles.

We selected endemic study sites in Kenya, Ethiopia and Sudan (Figure 1A) with stable VL transmission (Figure 1B) and distinct ecological features common to several VL foci in East Africa. Marsabit county in Kenya experienced sporadic outbreaks^(36, 37)^, reporting an incidence of 1 case per 10,0000 inhabitants over the past seven years excluding a notable increase in incidence during 2019. The Konso zone in Ethiopia, reported 2.66 cases per 10,000 inhabitants per year since 2018. In Sudan, Gedaref state is a highly endemic VL region that borders Ethiopia to the east and reports a steady 10 cases per 10,000 inhabitants per year since 2017 (Figure 1B).

### Vector species have distinct microhabitat and breeding preferences

**Figure 2.**
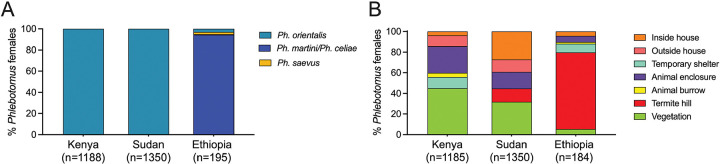
Distribution of different sand fly species across distinct microhabitats in endemic foci in Kenya, Sudan and Ethiopia study sites (A) Relative abundance of collected *Phlebotomus* females by species. (B) Relative abundance of *Phlebotomus* females by ecotype.

Entomological surveys of phlebotomine sand flies were carried out periodically from September 2023 to September 2024 (Supplementary Table 1). Trapping sites were selected from representative peridomestic and sylvatic ecotypes (Supplementary Figure 1). Our collections determined that *Ph. orientalis* was the predominant VL vector species in Marsabit, Kenya, and Gedaref, Sudan, comprising 99.75% (n = 1185) and 100% (n = 1350) of *Phlebotomus* females, respectively (Figure 2A). In contrast, *Ph. martini/Ph. celiae* females (morphologically indistinguishable) were the most prevalent of five *Phlebotomus* species in Konso, Ethiopia, accounting for 99% of the collection (n = 184; Figure 2A). Other female *Phlebotomus* species captured included one *Ph. martini/Ph. celiae* and two *Ph. alexandri* in Kenya; and six *Ph. orientalis*, four *Ph. saevus,* and one *Ph. alexandri* in Ethiopia.

Comparing ecotype productivity of *Ph. orientalis* and *Ph. martini/Ph. celiae* revealed a significant difference in habitat distribution across all study sites in the three countries (c^2^ = 1352.89, d.f. = 12, *P* <0.0001). The majority of *Ph. orientalis* females in Marsabit, Kenya, were collected from outdoor habitats associated with peridomestic and sylvatic vegetation (45.01%, *P* <0.0001), mostly *Acacia* and *Balanites* trees, followed by animal enclosures (25.92%, *P* <0.0001; Figure 2B). In Gedaref, Sudan, *Ph. orientalis* was primarily captured in vegetation (31.78%, *P* <0.0001) and inside houses (27.19%, *P* <0.0001) whose floors commonly consist of black cotton soil, a risk factor for VL^(19, 24)^. The remaining specimens were collected from outside houses (12.15%) and in animal enclosures (15.93%; Figure 2B). Worth noting, the majority of *Ph. orientalis* females (39.34%) were collected near human dwellings. In Konso, Ethiopia, *Ph. martini/Ph. celiae* females were mainly captured from termite hills (74.46%, *P* <0.0001) that were prevalent in both peridomestic and sylvatic ecotypes, followed by temporary shelters locally known as “foras” (8.15%; Figure 2B).

We used *Phlebotomus* males to verify species prevalence and to indicate breeding sites. *Ph. orientalis* represented 100% of all collected males in Kenya (n = 260) and Sudan (n = 4714) (Supplementary Figure 2A). Like females, *Ph. orientalis* males in Marsabit, Kenya, were collected from vegetation (46.92%, *P* <0.0001), outside houses near *Acacia* and *Balanites* trees (18.85%), and in animal enclosures (19.23%) (Supplementary Figure 2B). Preliminary evidence of breeding sites, identified by finding unrotated or partially rotated male genitalia (n = 12), implicated outdoor ecotypes (Supplementary Figure 2C). In Gedaref, Sudan, *Ph. orientalis* males were mainly collected from vegetation (33.64%, *P* <0.0001) and indoors (22.70%, *P* <0.0001) (Supplementary Figure 2B), similar to females. Of the captured males with unrotated or partially rotated genitalia (n = 33), 51.52% (*P* <0.0001) were collected from vegetation and 21.21% from animal enclosures (Supplementary Figure 2C), potentially indicating that *Ph. orientalis* may not be breeding indoors in either Kenya or Sudan.

In Konso, Ethiopia, *Ph. martini* was the main male species collected (Supplementary Figure 2A), mostly from termite hills (72.35%, *P* <0.0001) (Supplementary Figure 2B), again similar to females. Other male species included *Ph. celiae* (5.80%) and *Ph. orientalis* (1.02%) (Supplementary Figure 2A). Some 72.92% (n = 35, *P* <0.0001) of *Ph. martini* males with unrotated or partially rotated genitalia were collected from termite hills (Supplementary Figure 2C), implicating this microhabitat as a significant breeding site for *Ph. martini*. Of note, we collected one *Ph. celiae* male with unrotated/partially rotated genitalia from a domestic animal enclosure. The relative abundance of *Ph. martini* over *Ph. celiae* males (94.14%) indicates predominance of the former species in our female collections.

### Vector species exhibit different feeding preferences

**Figure 3.**
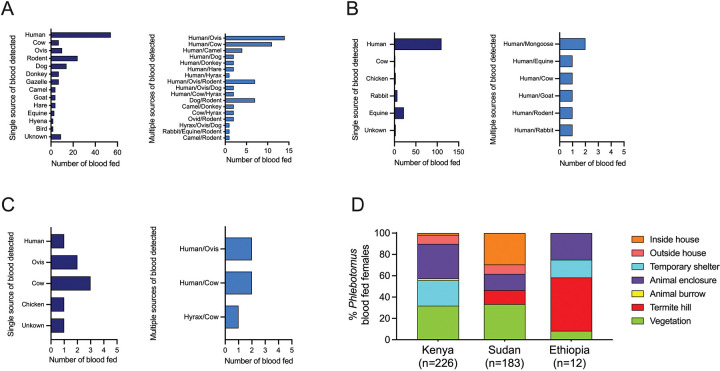
A mosaic of blood meal sources observed in leishmaniasis vectors across endemic foci in Kenya, Sudan and Ethiopia study sites (A-C) Host blood meal identity of blood fed females in Kenya (A), Sudan (B), and Ethiopia (C) with a single or multiple sources of blood detected. (D) Relative abundance of collected blood fed *Phlebotomus* females by ecotype.

We performed host blood meal analysis on field-collected individual blood fed *Phlebotomus* females using a custom-made multiplex PCR tailored to amplify blood from humans and nine animals abundant in our study sites (Supplementary Figure 3, Supplementary Table 2). The multiplex PCR panel identified 63.8% (n = 270/423) of blood meals. After *cytochrome b* (*cytb*) gene sequencing of unknowns this increased to 96.9% (n = 410/423). Interestingly, the diversity of identified blood meal sources was considerably higher in Marsabit, Kenya, (13 hosts) compared to either Gedaref, Sudan, (5 hosts) or Konso, Ethiopia, (4 hosts) (Figure 3A–C). Of interest, the observed number of sand flies with multiple blood meals were higher than expected for Marsabit, Kenya, (c^2^ = 44.09, d.f. = 15, *P* <0.0001) and Gedaref, Sudan (c^2^ = 17.83, d.f. = 6, *P* <0.0067). Our results also establish that several females fed on humans and domestic or sylvatic animals such as hyraxes and mongooses (Figure 3A–C).

In Marsabit, Kenya, we collected 226 *Ph. orientalis* and two *Ph. alexandri* blood fed females from animal enclosures (32.30%, *P* <0.0001), vegetation (31.86%, *P* <0.0001), and temporary shelters (23.89%, *P* = 0.001), following a similar pattern to unfed/gravid sand flies (Figure 3D). Humans were identified as the dominant host for *Ph. orientalis*, representing 37.76% (*P* <0.0001) of single blood meals, followed by rodents (15.89%, *P* <0.0001), dogs (9.27%) and other sylvatic animals like gazelles (4.64%) (Figure 3A). Mixed blood meals from two or three hosts were identified in 63 *Ph. orientalis* specimens including human/ovis (22.22%), and human/cow (17.46%) (Figure 3A). In Gedaref, Sudan, 29.51% (*P* <0.0001) of the 183 collected blood fed *Ph. orientalis* specimens were captured inside the house, and 33.33% (*P* <0.0001) in vegetation (Figure 3D). Similar to Marsabit, Kenya, humans (74.83%, *P* <0.0001) were the predominant host, while 14.97% fed on donkey (Figure 3B). Only seven specimens contained mixed blood meals and fed on human plus other domestic and sylvatic animals such as mongooses and rabbits (Figure 3B). In Ethiopia, only 12 *Ph. martini/Ph. celiae* blood fed specimens were collected from termite hills (50%, *P* <0.001) and animal enclosures (25%) (Figure 3D). Contrary to the anthropophilic feeding behavior observed for *Ph. orientalis*, *Ph. martini/Ph. celiae* seemed to preferentially feed on cows (37.5%), and ovis (25.0%), and five sand flies fed on multiple hosts such as human/cow and hyrax/cow (Figure 3C). Of note, the two *Ph. alexandri* collected from Kenya fed on humans, while in Ethiopia, one *Ph. alexandri* fed on an Ovis.

### *Leishmania*-infected vector species were recovered from diverse microhabitats

**Figure 4.**
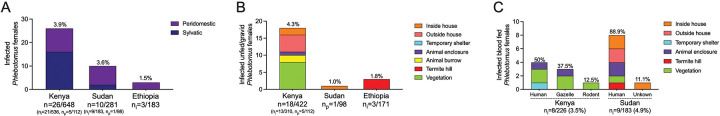
Distribution of *Leishmania*-infected sand flies from distinct and diverse microhabitats reveals hotspots of active transmission near and away human habitation (A) Number of *Leishmania*-infected sand flies collected from peridomestic or sylvatic habitats. (B) Habitat productivity of infected unfed/gravid female *Ph. orientalis* sand flies in Kenya and Sudan, and *Ph. martini/Ph. celiae* in Ethiopia. (C) Host blood meal identity of infected blood fed *Ph. orientalis and Ph. martini/Ph. celiae* females. Specimens were processed individually or as pools of up to 5–10 sand flies (B) or individually (C).

Using qPCR (Supplemental Figure 4) on individual (n_i_) or pooled (n_p_) sand flies, we detected infected specimens in both peridomestic and sylvatic niches across all study sites (Figure 4A), and from distinct ecotypes (Figure 4B–C). The minimal infection rates of *Ph. orientalis* with *Leishmania* were similar in Marsabit, Kenya, (26/648, 3.9%) and Gedaref, Sudan, (10/281, 3.6%). Ethiopia had a lower rate of infection of 1.5% (3/183) for *Ph. martini/Ph. celiae* females (Figure 4A). Of note, none of the other sand fly species collected throughout this study were infected.

In Marsabit, Kenya, 4.3% (18/422) unfed/gravid and 3.5% (8/226) blood fed *Ph. orientalis* were *Leishmania* positive (Figure 4B–C). Infected *Ph. orientalis* were associated with vegetation (30.77%), where we captured ten infected unfed/gravid and four infected blood fed females in sylvatic sites, and seven unfed/gravid and three blood fed *Ph. orientalis* from peridomestic sites (Supplementary Tables 1 and 3). Interestingly, a cluster of six infected sand flies was collected outside and inside the house of a relapsed VL patient in Laisamis, Kenya (Supplementary Tables 1 and 3, alluding to how hotbeds of transmission form. The patient, negative for the human immunodeficiency virus, received treatment in 2021 and refused treatment after relapsing in January 2024 (Supplementary Table 3), highlighting the need for better surveillance and treatment. Though four of the eight (50%) infected blood fed *Ph. orientalis* fed on humans, confirming the vector’s anthropophilic behavior, finding three and one infected sand flies that fed on gazelles and a rodent, respectively (Figure 4C, Supplementary Tables 3 and 4), stresses the need to investigate whether there is a role for animal reservoirs in VL transmission in East Africa.

For Gedaref, Sudan, one of the 98 (1.0%) screened unfed/gravid specimens collected from inside a house was positive for *Leishmania* (Figure 4B). Importantly, we found nine (9/183, 4.9%) blood fed *Leishmania*-infected *Ph. orientalis* females, seven from peridomestic sites, and two from vegetation and a termite hill in sylvatic sites within Dinder Park (Figure 4C, Supplementary Table 3). All nine infected sand flies fed on humans, further emphasizing the anthropophilic behavior of *Ph. orientalis* in this region (Figure 4C, Supplementary Tables 3 and 4). The focal nature of transmission was again revealed by finding three infected blood fed sand flies, two inside the house of a VL patient, collected in consecutive days, and a third that was captured from inside the house of the patient’s neighbor (Supplementary Tables 1 and 3).

In Ethiopia, three (1.8%, n=3/171) unfed/gravid *Ph. martini/Ph. celiae* were positive for *Leishmania,* all captured in termite hills (Figure 4B). Two of the three were captured 9 months apart from the same castellated termite hill located in Galga near the house of a recent VL case (Supplementary Tables 1 and 3 ), again highlighting the focality of VL. The prevalence of *Ph. martini/Ph. celiae* females in termite hills and the exclusive collection of *Leishmania*-infected specimens from this microhabitat implicates it in VL transmission in Ethiopia.

### *Leishmania* rK39 seroprevalence reveals an increased risk of infection for individuals living near VL cases

As *Ph. orientalis* is the vector of *L. donovani* in both Marsabit, Kenya, and Gedaref, Sudan, we wanted to assess seroprevalence of *Leishmania* rK39 antibodies in these study foci. We conducted a case-control cross-sectional survey in household members of VL cases and five neighboring households for comparison against controls (households located >4000 meters from confirmed cases). DBSs were collected from household members of 48 VL cases diagnosed during December 2023 to May 2025 in Laisamis and Karare villages, Marsabit county, Kenya, and from five VL cases diagnosed during August 2023 to August 2024 in Kunaynah village, Gedaref state, Sudan (Supplementary Table 5). In Kenya, we enrolled 752 subjects (male to female ratio of 1:1.54) with a median age of 14 years (range, 2 – 85 years). In Sudan, we enrolled 440 participants (male to female ratio of 1:1.3) with a median age of 15 years (range, 5 months – 85 years). Thirty-two and ten control households were screened from control HHs in Kenya and Sudan, respectively.

**Figure 5.**
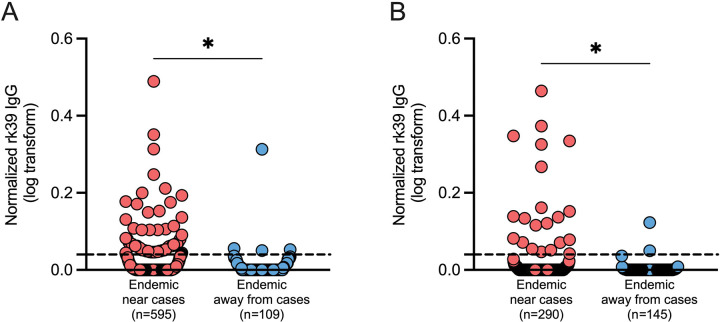
*Leishmania* rK39 antibody prevalence in our study sites indicate high risk of infection in Marsabit county, Kenya, and Gedaref state, Sudan (A,B) *Leishmania* rK39 antibody prevalence measured from dry blood spots (DBS) collected from individuals living with or near and away recently reported VL cases in Laisamis and Karare, Marsabit county (A) and Kunaynah, Gedaref state (B). Data was normalized using the plate cutoff calculated by the mean optical density (O.D.) of non-endemic negative controls + 2 SD. Non-endemic negative controls were obtained from individuals living in Nairobi, Kenya. Black dotted line, samples with O.D. ≤ 0.04 were considered negative after normalization. A *P* value of ≤ 0.05 was considered significant by Mann-Whitney test (A,B); *P < 0.05.

The rK39 titers of individuals living near cases were significantly higher than those of control households in both Kenya and Sudan (Figure 5). In Kenya, 21.68% (129/595) of screened asymptomatic participants living with or near VL cases exhibited significantly higher rK39 antibodies, compared to 16.51% (18/109) of those living farther away (Figure 5A). Similarly, 10.03% (29/289) of asymptomatic people living with or near VL cases in Sudan were seropositive, versus only 2.05% (3/146) of those living away from cases (Figure 5B). Of note, at the time of serum collection, only 23/48 (Kenya) and 2/5 (Sudan) of the diagnosed and treated VL patients had detectable antibodies by rK39 ELISA (Supplementary Table 5), considered the gold standard VL serodiagnostic test, even though DBSs were collected within 1–4 months of treatment.

## Discussion

The WHO has recently targeted visceral leishmaniasis in East Africa for elimination as a public health concern by 2030^(11)^. In the present study, we provide a comprehensive account of sand fly species composition, habitat preference, and *Leishmania*-infection rates within distinct VL endemic foci in three East African countries. We also compare the prevalence of asymptomatic cases near and away from recently treated VL cases in endemic foci. Our findings provide evidence of widespread and intense VL transmission in diverse settings across our study sites.

Consistent with earlier reports from the region^(6, 8, 49)^, *Ph. orientalis* was the most prevalent *Phlebotomus* species in Sudan and northern Kenya, while *Ph. martini* predominated in southern Ethiopia. *Ph. orientalis* was predominant in *Acacia* and *Balanites* forests in association with vertisol and animal burrows, microhabitats reported to be suitable breeding sites for this vector^(8, 17, 20, 50)^. In comparison, termite hills were the most productive site for *Ph. martini*, reported previously^(7, 51–53)^, though the collection of over 72% of newly emerged males from this microhabitat provides evidence of its significance as a breeding site.

Several studies have reported infection rates in field-collected specimens from Kenya (0.3%)^(54)^, Sudan (1.6% and 1.4%)^(19, 55)^, and southern (5.8%)^(7)^ and northern Ethiopia (23%)^(23)^. In our study, the infection rates were higher than previously reported for Kenya (3.9%) and Sudan (3.6%) and lower for southern Ethiopia (1.5%). More studies targeted towards establishing the longevity, population structure, and feeding behavior of vectors from field-captured specimens, particularly for *Leishmania*-infected females, are needed to fully understand vectorial competence and VL transmission in East Africa.

We recovered *Leishmania*-infected *Ph. orientalis* from different microhabitats within our study sites in Marsabit, Kenya, and Gedaref, Sudan, in both sylvatic and peridomestic niches, and frequently from the same location months apart. This indicates that *Ph. orientalis* has adapted to diverse ecological microhabitats that drive focal transmission of VL by providing sugar and blood meal sources, and breeding habitats^(17, 18, 20)^. In addition, *Ph. orientalis* likely shifts its behavior to adapt to climatic conditions. Collections from Helat-Belo, Gedaref state, Sudan, conducted during the dry season in March to June 2016–2018 found infected blood fed *Ph. orientalis* mainly outside houses and in sylvatic ecotypes, and reported that they fed on human, cattle, and donkeys^(19)^. In contrast, our sand fly collections from Kunaynah and Umslala-Houg were performed during the rainy season (June to September), when people tend to sleep inside their houses. Consequently, *Leishmania*-infected *Ph. orientalis* females were collected mainly indoors and had fed soley on humans, suggesting a potential shift in vector behavior, with sand flies likely resting inside the house after feeding on humans. While the majority of juvenile *Ph. orientalis* males were collected from vegetation and animal enclosures, we also collected juvenile males indoors in Sudan, possibly due to the widespread nature of black cotton soil in the village, including inside houses, serving as a breeding site for this vector^(20)^, and creating an ideal environment for focal and active VL transmission indoors. Further confirmation of this shift in vector behavior and transmission pattern is needed to inform control measures that are currently focused on outdoor applications.

In Konso, Ethiopia, *Ph. martini/Ph. celiae* were predominantly collected from termite hills, both in sylvatic and peridomestic settings, pointing to a restricted habitat preference compared to *Ph. orientalis*. Additionally, all three *Leishmania*-infected sand flies were recovered from termite hills reinforcing findings of a previous study^(7)^. Capturing two *Leishmania*-infected females nine months apart from a peridomestic castellated termite hill reinforces the focality of infection sources and highlights the importance of this microhabitat in sustaining transmission by *Ph. martini/Ph. celiae*. Notably, this termite hill was located only 20 meters away from a household that reported a VL case.

Finding *Leishmania*-infected sand flies near VL cases from all our study sites affirms the importance of human reservoirs in maintaining VL transmission, and the need for continued surveillance and rapid treatment of cases. This is further reinforced by rK39 serological surveys where participants living near VL cases exhibited significantly higher antibody levels compared to those living farther away from cases in both Marsabit, Kenya, and Gedaref, Sudan, highlighting the risk associated with proximity to VL cases. Overall, the high prevalence of rK39 antibodies in asymptomatic participants provides evidence of prevalent and efficient transmission, and stresses the urgency of determining their involvement as potential parasite reservoirs^(56)^.

Our study and others^(7, 19, 21, 23, 54, 55)^ have reported infected sand flies from both sylvatic and domestic/peri-domestic habitats. Host attractiveness to vector sand flies, seroprevalence of *Leishmania* antibodies, and PCR detection of *L. donovani* DNA have been reported for various domestic and wild animal species in East Africa^(27, 28, 57–59)^, but live parasites have not been isolated from any thus far. Irrespective of whether domestic or wild animals are reservoirs, VL endemicity in relatively scarcely populated areas points to the existence of a zoonotic transmission cycle in East Africa^(19, 24, 60)^. Establishing whether animal reservoirs are involved in sustaining VL transmission in East Africa is a major knowledge gap that needs to be urgently addressed to inform on the best strategy for VL elimination in this region.

A limitation of our study is the use rK39 to assess seroprevalence. The sensitivity of rK39 is East Africa is suboptimal^(14–16)^. Diagnostic tools with better specificity and sensitivity to detect *Leishmania* infections in East Africa are needed. For studies of vector sand flies, our aim was to identify their preferred habitats through a broad investigation of distinct foci within three East African countries. Moving forward, in-depth longitudinal studies focused on identified hotbeds of infection will be needed to better understand transmission dynamics throughout the season. Another study limitation is the pooling of non-fed specimens which provided a minimal infection rate. A high throughput in-depth analysis of infection status in individual specimens, conducted in defined microhabitats, will yield original data vital to elucidation of vector competence in VL foci in East Africa.

Our exploratory ecoepidemiological study conducted in three East African countries reinforces widespread and intense VL transmission in the region. Our results also pinpoint habitat preference of vector species and transmission hotspots in distinct microhabitats within endemic foci that emphasize both the focality and complexity of VL transmission in East Africa. Concurrent longitudinal clinical and entomological studies of several disease foci throughout East Africa are needed to galvanize an effective elimination campaign.

## Supplementary Material

Supplement 1

## Data Availability

All data produced in the present work are contained in the manuscript
