# Supplementary material for: Widespread transmission in diverse ecotypes challenges visceral leishmaniasis control in East Africa
Source: medRxiv. 2025 Nov 24:2025.10.01.25337091. Originally published 2025 Oct 3. Preprint. [Version 2] doi: 10.1101/2025.10.01.25337091 (PMC12622091; doi:10.1101/2025.10.01.25337091)
Supplement: Supplement 1 [file media-1.pdf]

Supplementary information

Widespread transmission in diverse ecotypes challenges visceral leishmaniasis control in East Africa

Eva Iniguez[1†*], Mercy Tuluso[2†], Steve Kiplagat[3], Araya Gebresilassie[4], Esayas Aklilu[5], Olivia Battistoni[6], Johnstone Ingonga[2], John Mark Makwatta[3], Mohamed Alamin[7], Osman Dakien[7,8], Alphine Chebet[2], Esther Kaunda[2], Patrick Huffcutt[1], Serena Doh[1], Pedro Cecilio[9], Galgallo Bonaya[3], Claudio Meneses[1], Tiago D. Serafim[1], Myrthe Pareyn[10], Mohamed Osman[11], Eltahir A. G. Khalil[7], Omran F. Osman[7], Brima M. Younis[7], Sithar Dorjee[12], Guofa Zhou[13], Jesus G. Valenzuela[1], Dan K. Masiga[3], Ahmed M. Musa[14], Asrat Hailu[15], Abhay Satoskar[6], Shaden Kamhawi[1*], Damaris Matoke-Muhia[2*]

†Equal contribution

*Corresponding authors: Eva Iniguez; Vector Molecular Biology Section, Laboratory of Malaria and Vector Research, National Institute of Allergy and Infectious Diseases, National Institutes of Health; Rockville, MD 20852, USA.; Phone: 1.301.761.7357; Email: eva.iniguez@nih.gov. Shaden Kamhawi; Vector Molecular Biology Section, Laboratory of Malaria and Vector Research, National Institute of Allergy and Infectious Diseases, National Institutes of Health; Rockville, MD 20852, USA.; Phone: 1.301.761.5081; Email: skamhawi@niaid.nih.gov. Damaris Matoke-Muhia; Centre for Biotechnology Research and Development, Kenya Medical Research Institute, Nairobi 00200, Kenya; Phone: 54.722.205.901; Email: DMatoke@kemri.go.ke.

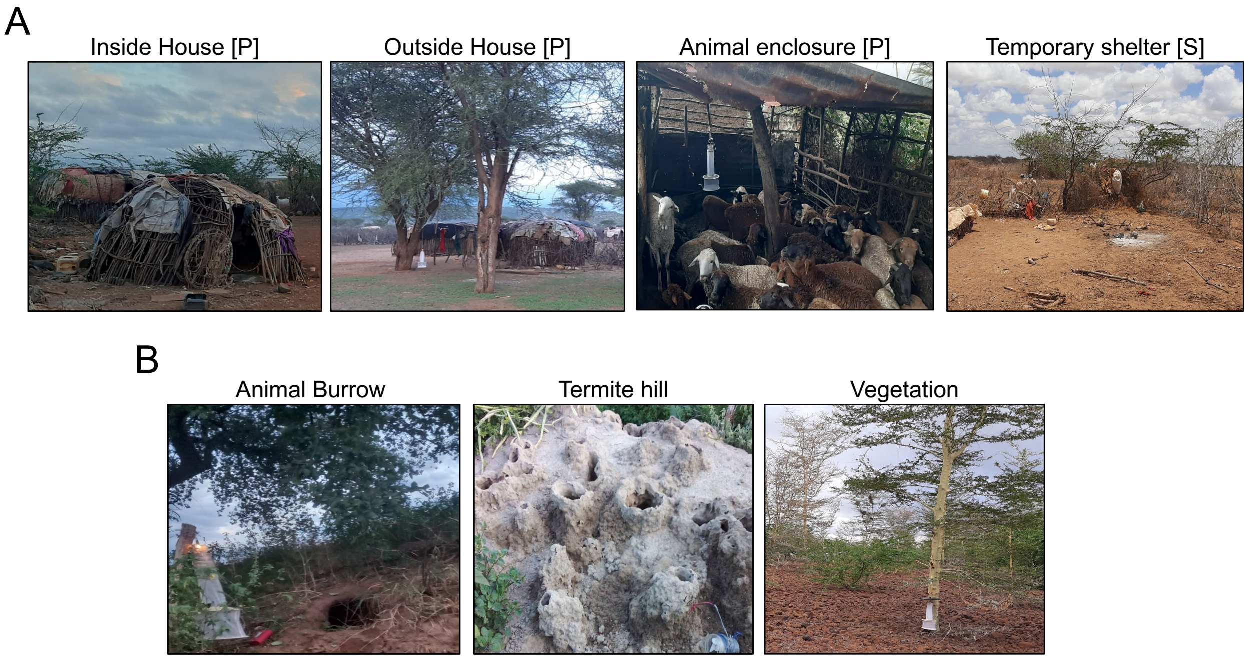

Supplemental Figure 1. Peridomestic and sylvatic sand fly trapping ecotypes
(A) Representative images of ecotypes present in peridomestic (P) or sylvatic (S) sites. (B) Representative images of ecotypes present in both peridomestic and sylvatic sites.

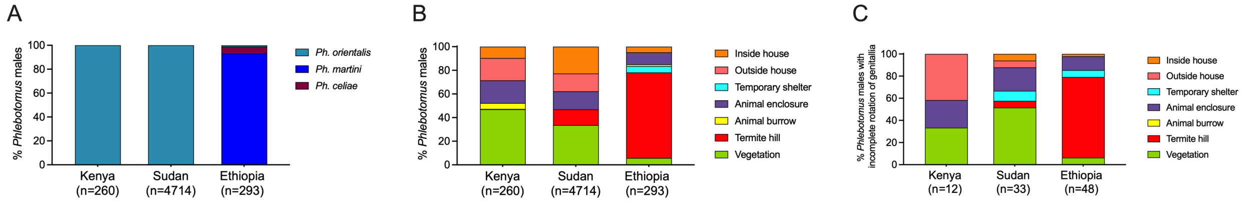

Supplementary Figure 2. Males reveal the breeding microhabitats of VL vectors in East Africa

(A) Relative abundance of collected *Phlebotomus* males by species. (B) Relative abundance of *Phlebotomus* males by ecotype. (C) Breeding habitat productivity based on finding unrotated/partially rotated male genitalia.

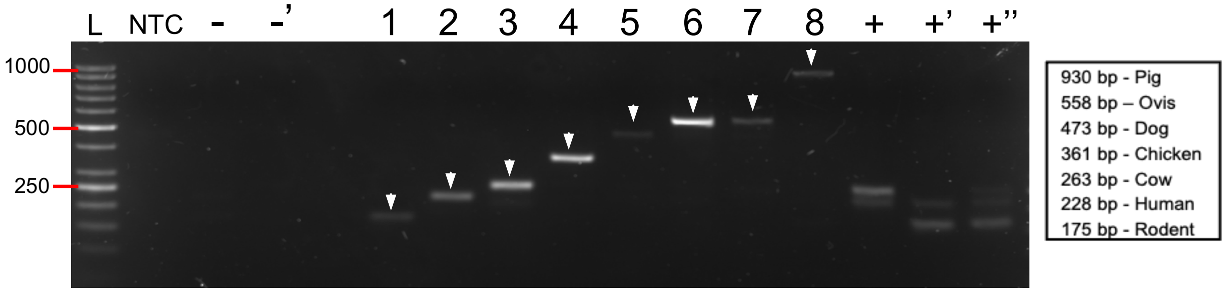

Supplementary Figure 3. Representative PCR panel for multiplex blood meal analysis. Multiplex PCR panel for blood meal analysis. Gel shows bands of distinct sizes to distinguish human and seven common vertebrate hosts in East Africa. 1, rodent; 2, human; 3, cow; 4,chicken; 5, dog; 6, goat (Ovis); 7, sheep (Ovis); 8, pig. Negative controls, DNA from unfed sand fly (-), or from an experimental sand fly fed on rabbit blood absent in this panel (-’); Positive controls, DNA from an experimental sand fly fed on a mixed cow/human (+), rodent/human (+’), rodent/cow/human (+”) blood meals. L, ladder. NTC, non-template control. Arrowhead, single blood meal. Rectangles, mixed blood meals.

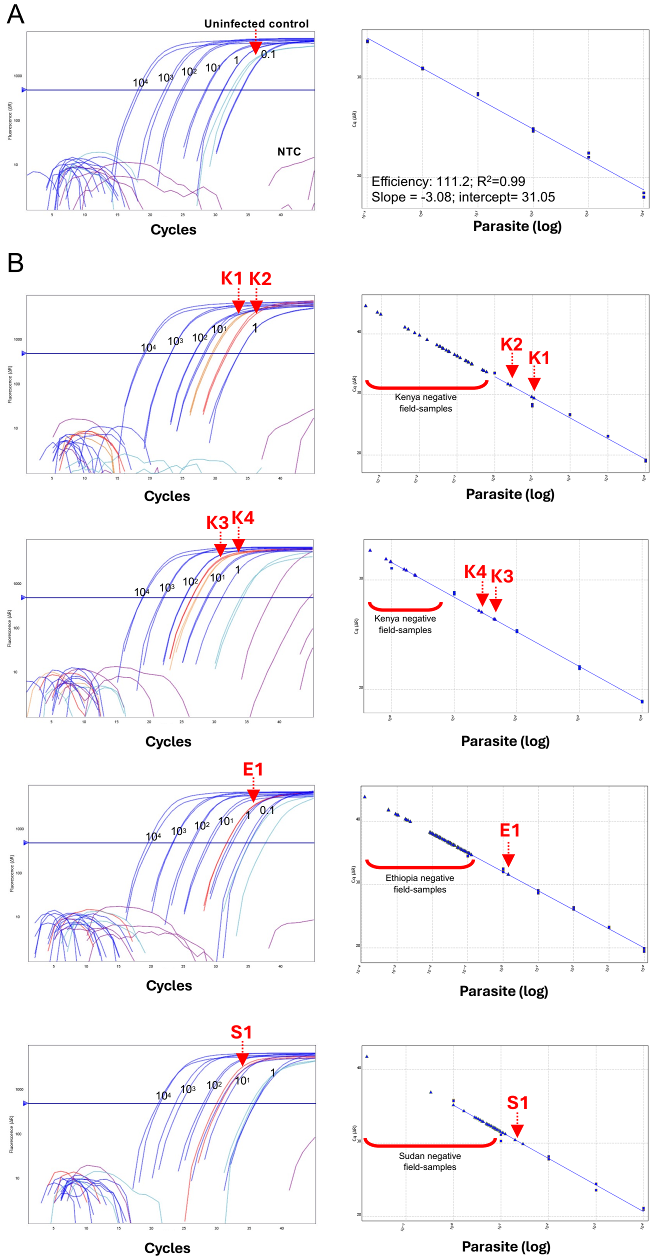

Supplementary Figure 4. *Leishmania* qPCR on field-collected *Phlebotomus* sand flies
(A) qPCR standard curve. (B) Representative qPCR amplification plots of positive *Leishmania*-infected field-collected samples from Kenya, Ethiopia, and Sudan. K, Kenya; E, Ethiopia; S, Sudan. Dark blue, standard curve from 10^4 to 1 *Leishmania donovani* parasites spiked in one uninfected sand fly midgut (Kenya and Ethiopia) or whole body (Sudan); Aqua, uninfected negative sand fly midgut; purple, non-template control; Red or orange, field samples.

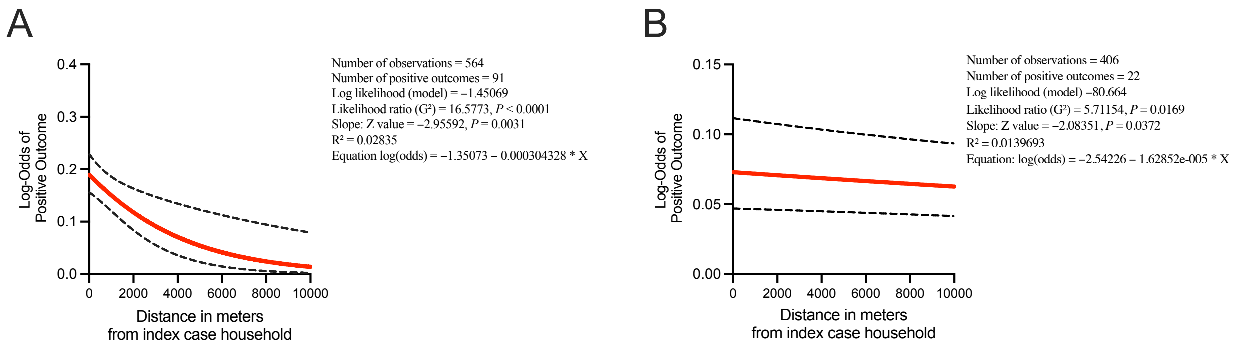

Supplementary Figure 5. Effect of distance from index case household on rK39 seropositivity. (A-B) Univariable binary logistic regression analyses was performed to assess the effect of distance (meters) from index case households to neighboring or control households, in correlation with rK39 positivity after normalization in Marsabit, Kenya (A), and Gedaref, Sudan (B). The ‘margins’ command of Stata was used to compute adjusted log-odds of seropositivity at selected distances from the index households. Analysis does not include index household members.

Supplementary Table 1. Trapping sites and focality of *Leishmania*-infected *Phlebotomus* sand flies.

Country	Study site	Date of collection	Per trip			
			Average no. of traps per day	No. of traps with infected specimens	No. of infected specimens per trap	Trap ID
Marsabit Kenya	Laisamis	January, 2024	20	0	0	-
		April, 2024	20	4	1_i, 1_i, 1_i, 3_i	2, 10, 5, 4
		August, 2024	20	0	0	-
	Log logo	January, 2024	20	0	0	-
		March, 2024	20	3	1_i, 1_i, 2_i, 1_i, 1_i	**3**, 8, **7, 7, 3**
		July, 2024	20	10	3_p+1_i, 1_p, 1_i, 1_i, 1_i, 1_i, 1_i, 1_i, 1_i, 1_i, 1_p,	19, 1, 17, 13, **2**, 7, **2**, 21, 22, 11, 5
		August, 2024	20	0	0	-
Gedaref state Sudan	Kunaynah	September, 2023	12	0	0	-
		October, 2023	12	0	0	-
		December, 2023	12	2	1_i, 1_i	5, 7
		January, 2024	12	2	1_p, 1_i	10, 8
		September, 2024	12	0	0	
	Umslala-Houng and Dinder Park	November, 2023	12	0	0	-
		April, 2024	12	0	0	-
		June, 2024	12	5	1_i, 2_i, 1_i, 1_i, 1_i	6, 7, 10, 8, 12
Konso Aba-Roba Ethiopia	Galga	September, 2023	9.5	1	1_i	**14**
		November, 2023	9.5	0	0	-
		February, 2024	12	0	0	-
		June, 2024	15	2	1_i, 1_i	**14**, 15
	Maira	September, 2023	7	0	0	-
		November, 2023	10.33	0	0	-
		February, 2024	11	0	0	-
		June, 2024	15	0	0	-
	Lahalaha and Goinada	September, 2023	15	0	0	-
		November, 2023	10.33	0	0	-
		February, 2024	11	0	0	-

Specimens processed individually (i) or as a pool (p). D, day.Bold, trap IDs that appear more than once per study site.

Supplementary Table 2. Primer sequences and concentrations used in the custom-designed multiplex panel for host blood meal identification by PCR and for generic *cytb* gene amplification for sequencing.

Host-blood meal multiplex-PCR target species					
**Species**	**5'-3' Sequence**	**Band Size (bp)**	**Stock μM**	**Reaction μM**	**μl per primer for a 30μL reaction**
Mongoose Fwd*	GGTGAAACCTCGGCTCTCTT	937	10	0.183	0.55
Equine (donkey/horse) Fwd*	ACTGCCTTCTCATCCGTCAC	845	10	0.117	0.35
Chicken Fwd	CAGCAGACACATCCCTAGCC	723	10	0.042	0.125
Chicken Rev	AGGCTGCTAGGGCTAGTACA	723	10	0.042	0.125
Camel Fwd*	GGCTACGTCCTTCCATGAGG	638	10	0.033	0.1
Ovis (goat/sheep) Fwd*	TGGCACAAACCTAGTCGAATGA	558	10	0.067	0.2
Dog Fwd*	CTCCCTTTCATCATCGCAGCTC	473	10	0.05	0.15
Hyrax Fwd*	CCAACGCCGACAAAATCCCAT	388	10	0.133	0.4
Cow Fwd*	AACTACACCCCAGCCAATCC	263	10	0.05	0.15
Human Fwd[42]	TTCGGCGCATGAGCTGGAGTCC	228	10	0.067	0.2
Human Rev[42]	TATGCGGGGAAACGCCATATCG	228	10	0.067	0.2
Muroidea Fwd[43]	TCTGGTTCTTACTTCAGGGC	175	20	0.5	0.75
Muroidea Rev[43]	TTCATGCCTTGACGGCTATG	175	20	0.5	0.75
Vertebrate UNRev[41]*	GGTTGTCCTCCAATTCATGTTA	-	10	0.267	0.8
Vertebrate AltRev*	GGCTGGCCTCCAATTCATGTGA	-	10	0.033	0.1
**Generic vertebrate *Cytb* primer sequence**					
CytB Fwd	CCATCAAACATCTCAGCATGATGAAA	358	10	0.5	1.25
CytB Rev	CCCCTCAGAATGATATTTGTCCTC	358	10	0.5	1.25

*Forward primer for use with listed universal vertebrate reverse primer.

Supplementary Table 3. Date of collection and ecotype of infected *Phlebotomus* sand flies from our study sites.

Study site	Peridomestic /sylvatic	Ecotype	Date of collection	Physiological status, blood meal source
Log Logo, Kenya	Sylvatic	Vegetation	3/27/24	Unfed
		Vegatation; vertisols	3/28/24	Unfed
		Vegatation; vertisols	3/29/24	Unfed
		Vegatation; vertisols	3/29/24	Unfed
		Vegatation; vertisols	3/31/24	Unfed
		Vegetation	3/31/24	Unfed
		Vegetation	07/24/24	Blood fed, human
		Vegatation; vertisols	07/24/24	Blood fed, rodent
		Vegetation	07/24/24	Blood fed, human
		Vegatation; vertisols	07/24/24	Blood fed, Gazelle
		Vegatation; vertisols	07/24/24	Unfed pool
		Vegatation; vertisols	07/24/24	Unfed pool
		Vegatation; vertisols	07/24/24	Unfed pool
		Animal enclosure	07/24/24	Unfed pool
		Vegetation	07/24/24	Gravid
		Temporary shelther	07/26/24	Blood fed, human
	Peridomestic	Animal enclosure	07/26/24	Blood fed, human
		Outside house	07/26/24	Blood fed, Gazelle
		Animal enclosure	07/26/24	Blood fed, Gazelle
		Indoors	07/29/24	Unfed pool
Laisamis, Kenya	Peridomestic	Outside house of VL patient #2, Patient relapsed in January, 2024; no treatment received; HIV* negative.	4/10/24	Gravid
		Outside house of neighbor #5 of VL patient #2	4/10/24	Gravid
		Inside house of neighbor #3 of VL patient #2	4/10/24	Unfed
		Outside house of neighbor #2 of VL patient #2	4/11/24	Unfed
		Outside house of neighbor #2 of VL patient #2	4/11/24	Gravid
		Outside house of neighbor #2 of VL patient #2	4/11/24	Unfed
Kunaynah, Sudan	Peridomestic	Inside house of VL patient #3	12/15/23	Blood fed, human
		Inside house of VL patient #3's neighbor	12/16/23	Blood fed, human
		Inside house	1/7/24	Blood fed, unkown
		Inside house	1/7/24	Unfed pool
Umslala-Houg & Dinder Park, Sudan	Peridomestic	Outside house	6/7/24	Blood fed, human
		Animal enclosure	6/7/24	Blood fed, human
		Animal enclosure	6/7/24	Blood fed, human
		Outside house	6/7/24	Blood fed, human

	Sylvatic	Termite hill	06/11/24	Blood fed, human
		Vegetation	06/13/24	Blood fed, human
Galga, Ethiopia	Peridomestic	Termite hill	9/27/23	Unfed
		Termite hill	06/23/24	Gravid
		Termite hill	06/23/24	Gravid

* Human immunodeficiency virus.

Supplementary Table 4. Confirmation of blood meal host identification of *Leishmania* infected samples by cytochrome b (*cyt b*) gene sequencing. PCR amplification of vertebrate *cytb* gene using universal primer sets was carried out and the product was sequenced. BLAST results confirmed the host with exact or nearly exact matches by the percent identity. A bit E-score >50, coverage >35%, identity >70% were considered as valid.

Study site	Species by *Cyt b* sequencing	Query Cover primer	E-value	% Identity	Species
Log Logo, Kenya	Gazelle	79%	2.00E-118	92.46%	Gazelle
Log Logo, Kenya	Human	97%	9.00E-158	99.38%	Human
Log Logo, Kenya	Human	95%	1.00E-150	98.44%	Human
Log Logo, Kenya	Human	97%	7.00E-153	98.46%	Human/Dog
Log Logo, Kenya	Human	96%	1.00E-156	99.07%	Human
Log Logo, Kenya	Gazelle	83%	3.00E-134	93.94%	Gazelle
Log Logo, Kenya	Gazelle	93%	2.00E-110	89.81%	Gazelle
Log Logo, Kenya	-	-	-	-	Rodent
Dinder Park, Sudan	Human	85%	1.00E-151	97.55%	Human
Dinder Park, Sudan	Human	96%	4.00E-156	98.77%	Human
Umslala-Houg, Sudan	Human	97%	2.00E-154	98.46%	Human
Umslala-Houg, Sudan	Human	93%	6.00E-129	93.91%	Human
Umslala-Houg, Sudan	Human	97%	3.00E-158	99.38%	Human
Umslala-Houg, Sudan	Human	96%	1.00E-157	99.38%	Human
Kunaynah, Sudan	Human	68%	4.00E-149	98.69%	Human
Kunaynah, Sudan	Human	67%	2.00E-142	97.69%	Human
Kunaynah, Sudan	Uknown	-	-	-	Unkown

Supplementary Table 5. Dates of diagnosis and treatment and serum collection from VL patients in our study sites in Marsabit county, Kenya, and Gedaref state, Sudan.

Study site	Patient ID	Date of clinical diagnosis (rK39 RTD and /or microscopy) and treatment	This study Date of DBS collection	This study DBS rK39 ELISA
Laisamis, Marsabit Kenya	1	December, 2023	January, 2024	-
	2	First diagnosis and treatment in 2021. Patient relapsed in January, 2024; no treatment received; HIV* negative.	April, 2024	-
	3	February, 2024	April, 2024	-
Karare, Marsabit Kenya	4	July, 2024	August, 2024	+
	5	June, 2024	August, 2024	+
	6	June, 2024	August, 2024	-
	7	June, 2024	August, 2024	-
Laisamis, Marsabit Kenya	8	September, 2024	January, 2025	-
	9	September, 2024	January, 2025	-
	10	September, 2024	January, 2025	-
	11	October, 2024	January, 2025	-
	12	October, 2024	January, 2025	+
	13	November, 2024	January, 2025	-
	14	November, 2024	January, 2025	-
	15	November, 2024	February, 2025	+
	16	December, 2024	February, 2025	+
	17	December, 2024	February, 2025	+
	18	December, 2024	February, 2025	+
	19	December, 2024	February, 2025	-
	20	December, 2024	February, 2025	-
	21	January, 2025	February, 2025	-

Laisamis, Marsabit Kenya	22	January, 2025	February, 2025	+
	23	January, 2025	February, 2025	+
	24	January, 2025	February, 2025	+
	25	January, 2025	February, 2025	+
	26	February, 2025	February, 2025	-
	27	February, 2025	February, 2025	-
	28	January, 2025	May, 2025	+
	29	January, 2025	May, 2025	+
	30	February, 2025	May, 2025	-
	31	February, 2025	May, 2025	-
	32	February, 2025	May, 2025	+
	33	March, 2025	May, 2025	+
	34	March, 2025	May, 2025	-
	35	March, 2025	May, 2025	+
	36	March, 2025	May, 2025	-
	37	March, 2025	May, 2025	-
	38	March, 2025	May, 2025	+
	39	March, 2025	May, 2025	+
	40	March, 2025	May, 2025	-
	41	April, 2025	May, 2025	-
	42	April, 2025	May, 2025	+
	43	April, 2025	May, 2025	-
	44	April, 2025	May, 2025	+
	45	April, 2025	May, 2025	+
	46	April, 2025	May, 2025	+
	47	April, 2025	May, 2025	-
	48	May, 2025	May, 2025	-
	1	August, 2023	October, 2023	-

Kunaynah, Gedaref state Sudan	2	September, 2023	December, 2023	-
	3	November, 2023	December, 2023	-
	4	December, 2023	January, 2024	-
	5	August, 2024	September, 2024	-

* Human immunodeficiency virus.
